# The COVID-19 Pandemic and Ethics in Mexico Through a Gender Lens

**DOI:** 10.1007/s11673-020-10029-4

**Published:** 2020-08-25

**Authors:** Amaranta Manrique De Lara, María De Jesús Medina Arellano

**Affiliations:** Bioethics, Health and Biolaw Program, Instituto de Investigaciones Jurídicas, Universidad Nacional Autónoma de México, Circuito Mario de La Cueva s/n, Ciudad Universitaria, 04510 Mexico City, Mexico

**Keywords:** Domestic violence, Pandemic, Equity, Global health ethics, Mexico

## Abstract

In Mexico, significant ethical and social issues have been raised by the COVID-19 pandemic. Some of the most pressing issues are the extent of restrictive measures, the reciprocal duties to healthcare workers, the allocation of scarce resources, and the need for research. While policy and ethical frameworks are being developed to face these problems, the gender perspective has been largely overlooked in most of the issues at stake. Domestic violence is the most prevalent form of violence against women, which can be exacerbated during a pandemic: stress and economic uncertainty are triggers for abuse, and confinement limits access to support networks. Confinement also exacerbates the unfair distribution of unpaid labor, which is disproportionately assigned to women and girls, and highlights inequality in the overall labor market. Lack of security measures has resulted in attacks towards health workers, particularly female nurses, due to fear of contamination. Finally, resource results in lack of access to other health necessities, including sexual and reproductive health services. Research across all disciplines to face—and to learn from—this crisis should be done through a gender lens, because understanding the realities of women is essential to understand the pandemic’s true effects in Mexico and the world.

This year’s International Women’s Day was a historic occurrence in Mexico (El Universal 2020). Tens of thousands of women took to the streets on the eighth of March and then chose to vanish on the ninth (Averbuch 2020). Each day in its own way, the so-called 8M and 9M were meant to raise awareness about femicides and the foundation of structural violence against women on which they stand. Women all across the country sought to generate a widespread debate and called for active commitment from key stakeholders and decision-makers. But while they marched through the streets in Mexico, the rest of the world was beginning to become paralyzed by a virus which has claimed over 600,000 lives globally, confining individuals and families to their homes, and overwhelming already fragile health systems (World Health Organization 2020). And in this unprecedented situation, girls and women in Mexico find themselves caught between a rock and a hard place of two public health crises—the pandemic and gender-based violence—in a country where misogyny seems part of our cultural heritage (Htun and Jensenius forthcoming).

In the Mexican context, structural violence against women is normalized and spans every sphere of society. Even the written press has played a role in this normalization, particularly through femicide coverage (Tiscareño-García and Miranda-Villanueva 2020). That is why a gender perspective should be included when analyzing issues, to find solutions that do not exacerbate inequality. During this pandemic, a lot of significant ethical and social issues have been raised, such as: the extent of restrictive measures, the reciprocal duties to healthcare workers, the allocation of scarce resources, and the need for research (Palacios-González 2020). While policy and ethical frameworks are being developed to face these problems, here we will try to show how the gender perspective has been largely overlooked in practice during the pandemic response in Mexico. Understanding the realities of women is essential to understand the true effects of this pandemic. Similarly, we believe including feminist approaches to the many ethical and social issues would allow decision-makers in Mexico to reach more optimal solutions. This is because traditional approaches tend to be abstract and based on universal absolutes, which ignore the diverse realities of women’s experiences. Further, these absolutes tend to focus on individualistic principles like autonomy, ignoring other ethical principles like solidarity, compassion, and communitarian values which are essential (Medina-Arellano 2020). Indeed, solidarity has been made a cornerstone of the global pandemic response, but how this principle is translated to practice both internationally and within our borders necessitates the recognition of power dynamics, including the prevalence of gender inequality.

The first topic of discussion is the deployment of restrictive public health measures. Social distancing measures, such as shelter-in-place orders, have become essential to counteract the rapid spread of SARS-CoV-2. However, besides seeming incompatible with the socioeconomic reality in Mexico where many people live day-to-day (Padrón Innamorato, Gandini, and Navarrete 2017), isolation and confinement at home are extremely concerning for women’s safety. In our country, domestic violence is the most prevalent form of violence against women, also impacting the lives of children (Scolese et al. 2020). Further, a considerable number of femicides is perpetuated in family, couple, or friendship environments (Lara Olmos 2020). In fact, the rate of femicides has increased by 7.7 per cent in the period of January to June 2020 compared to 2019 (Urrutia and Jiménez 2020). As the data shows, at-home violence and violence against women in general can be exacerbated during a pandemic: stress and economic uncertainty are triggers for abuse, and confinement pulls women away from their existing support networks or makes it difficult for them to find new ones.

Ideally, public programmes would have been set in place to respond to these issues before the general population was asked to stay at home. There are indeed existing programmes, like hotlines for gender violence. However, we need to realize that those measures are not sufficient in the current context and ask questions like: How can I call for help if there is constant forced coexistence with my aggressor? Where should I go to find shelter without being exposed to contagion? How can I file a lawsuit, or follow-up on one, while staying at home? Who ensures that I can have access to justice when the government workforce is not at full capacity?

Developing mobile applications to serve as panic buttons could be an effective solution (Ministerio de las Mujeres & Géneros y Diversidad 2020). Importantly, access to mental health support should be fully guaranteed, since suicide rates, especially among married women, are intricately linked to conflict within the household (Beleche 2019). However, we must always remember that violence against women is an intersectional issue. For example, socioeconomic status is a contributing factor to domestic violence, and the women who need the most help might be the same women who have less access to technology; the same is true for other marginalized women, such as indigenous or racialized women. Therefore, to be effective, these tools must be part of more widespread, cohesive policy efforts, relying on interinstitutional cooperation among relevant agencies.

Another consequence of confinement is that it highlights the existing inequality, based on misogynistic stereotypes, in the distribution of unpaid labor (i.e. household chores and care) (Salgado-Galicia et al. 2020). This inequality was normalized when authorities stated that women at home would have no issue looking after the health of elders, affirming that men tend to be “more detached” (Morales, Miranda, and Villa y Caña 2020). During quarantine, most women will be responsible for: increased household chores; caring for children, including aid in schoolwork and education due to schools closing down; tending to family members with any physical or mental disability; caring for the elderly; and, in some cases, looking after family sick with COVID-19.

Importantly, we should also be thinking about how the unequal distribution of domestic chores will affect young girls and teenagers during confinement (Wang et al. 2020). It is likely that having to do school from home will force them to put their education aside, since they will be expected to help with chores or care for their siblings, for example. Girls have less access to education and less academic opportunities than boys as it is, and we need to start thinking about ways to avoid this reality being exacerbated in our country post-pandemic. In that sense, we think it is also important that more women who work in the pandemic relief are showcased, especially those in top positions, to serve as role models. It is sadly unsurprising, but very illustrative of the inequality in our country, that most of the spearheads giving press conferences and shown on media are male, partly explained by the persistence of male directors and executives at the National Institutes of Health in Mexico (Rivera-Romano et al. 2020).

On top of the difficult situation at home, a lot of women will also take on professional responsibilities. This is made harder in a labour market where already they deal with increased job insecurity—including lack of access to health insurance—and lower salaries. These situations once again highlight the importance of an intersectional perspective, since indigenous women and women living in poverty are disproportionally affected; for example, those who participate in paid but exploitative domestic labor (Rojas-García and Toledo Gonzáles 2017).

Regarding healthcare workers, an important ethical issue is the scope and limitations of their duties during the pandemic. They will unavoidably face increased risk of contracting COVID-19, but this is only acceptable when there are reciprocal obligations from the state. This includes providing sufficient resources like face masks and other protective gear, including strategies to prevent burnout syndrome which is more prevalent on female medical students (Miranda-Ackerman et al. 2019). Broader security measures should also be provided, since healthcare workers, particularly female nurses, have sadly been attacked for fear of contamination. It must be stressed that women make up most of the healthcare workforce at the frontlines (World Health Organization 2019); therefore, governments, should ensure access to care facilities and school aids for their children, as well as services to aid in the care of any other dependents. Similarly, even though it is expected that the health system will be overwhelmed, female health workers who are in special situations, such as maternity leave or those who are breastfeeding, should not be, under any circumstance, expected to risk exposure and should not face any penalties. Finally, the public debate should avoid using hierarchical language based on sexist stereotypes when speaking about the healthcare workforce—*médicos* (male doctors) and *enfermeras* (female nurses)—which contributes to existing power dynamics.

Another ethical issue relates to the distribution of scarce resources. The fair allocation of ventilators is a particularly hot topic at this time, and the ethical framework developed in Mexico appropriately included a gender perspective (Guía Bioética 2020). However, medical resources and health staff being diverted to face the crisis will also affect health beyond COVID-19. This issue has been internationally recognized by the release of a joint statement regarding the protection of sexual and reproductive health and rights of women during the pandemic response (Klasing 2020). While Mexico signed said statement, there is still more work to be done in practice. The concern arises partly because rates of obstetric violence in Mexico are already high (Calvo Aguilar, Torres Falcón, and Valdez Santiago 2020), and it took some time to issue a special protocol regarding access to healthcare for pregnant women before, during, and after childbirth to prevent COVID-19 infection (Secretaría de Salud 2020). Another concern emerges from the lack of access to family planning and contraception methods. In particular, access to emergency health services for victims of sexual violence, such as safe means to interrupt a pregnancy resulting from rape, which is a recognized right in all of Mexico (NOM-046-SSA2-2005).

The provision of abortion services must be ensured in a timely manner, given that these procedures cannot be postponed and should be considered urgent, especially when they are legally recognized rights. Indeed, in addition to the rape causal nationwide (NOM-046-SSA2-2005 2009; Ministry of Health, Mexico 2020), the decision to interrupt a pregnancy on any grounds has been recently decriminalized in the state of Oaxaca, and abortion services are constitutionally guaranteed for any reason by the public health system in Mexico City. Beyond any moral opposition to abortion, these services are at risk of being set aside because they are sometimes thought to only pertain to women, and women’s interests are considered secondary. Women’s right to health is linked to their rights to life, free development of personality, human dignity, and sexual and reproductive freedom. All of these constitute undisputed human rights which cannot be discarded by any measure of exceptionality, even in the face of scarcity in the health sector. Women’s independence is inextricably linked to control over their bodies and reproduction, and any argument to justify taking away that right is undeniably a form of discrimination against women, especially towards women already marginalized in Mexico due to racism and classism.

One final issue raised by the pandemic is the need for research. We affirm that focused research must be done with a gender perspective. This includes: biomedical research about any differential responses to treatment; public health research to look at the effects of resource deviation on women’s physical health and of confinement on women’s mental health; economic research to look at the impact on the female informal workforce; and social research to see whether more girls are made to drop out of school and how rates of violence respond to social distancing. These approaches to research will prove invaluable not only to face this pandemic but to have real-life data in place to inform decision-makers in the future.

Among everything that has happened, this pandemic has managed to highlight and exacerbate the existing inequalities and flaws in our social structure (Papp and Hersh 2020). Even though the 8th of March and the women’s march seem like another lifetime, the demands remain current. The topics we have discussed about women’s access to health, justice, and a life free of violence must be fundamental issues in any and every plan to face this pandemic. Yes, we are all worried about the pandemic and what is to come, but we must remember that the structural violence women face every day remains itself an unattended public health crisis. Indeed, while social distancing to reduce transmission, women are stuck at home with their aggressors. While the health system struggles to provide life-sustaining services, eleven women are still dying every day in this country just for being women (Xantomila 2020). The government must not consider this a secondary issue, because women in Mexico sure do not have the option of forgetting.
